# Supplementary material for: The liver-enriched lnc-LFAR1 promotes liver fibrosis by activating TGFβ and Notch pathways
Source: Nat Commun. 2017 Jul 26;8:144. doi: 10.1038/s41467-017-00204-4 (PMC5529527; doi:10.1038/s41467-017-00204-4)
Supplement: Supplementary file 1 — Supplementary Information [file 41467_2017_204_MOESM1_ESM.pdf]

File name: Supplementary Information
Description: Supplementary figures and supplementary tables.

File name: Peer review file
Description:

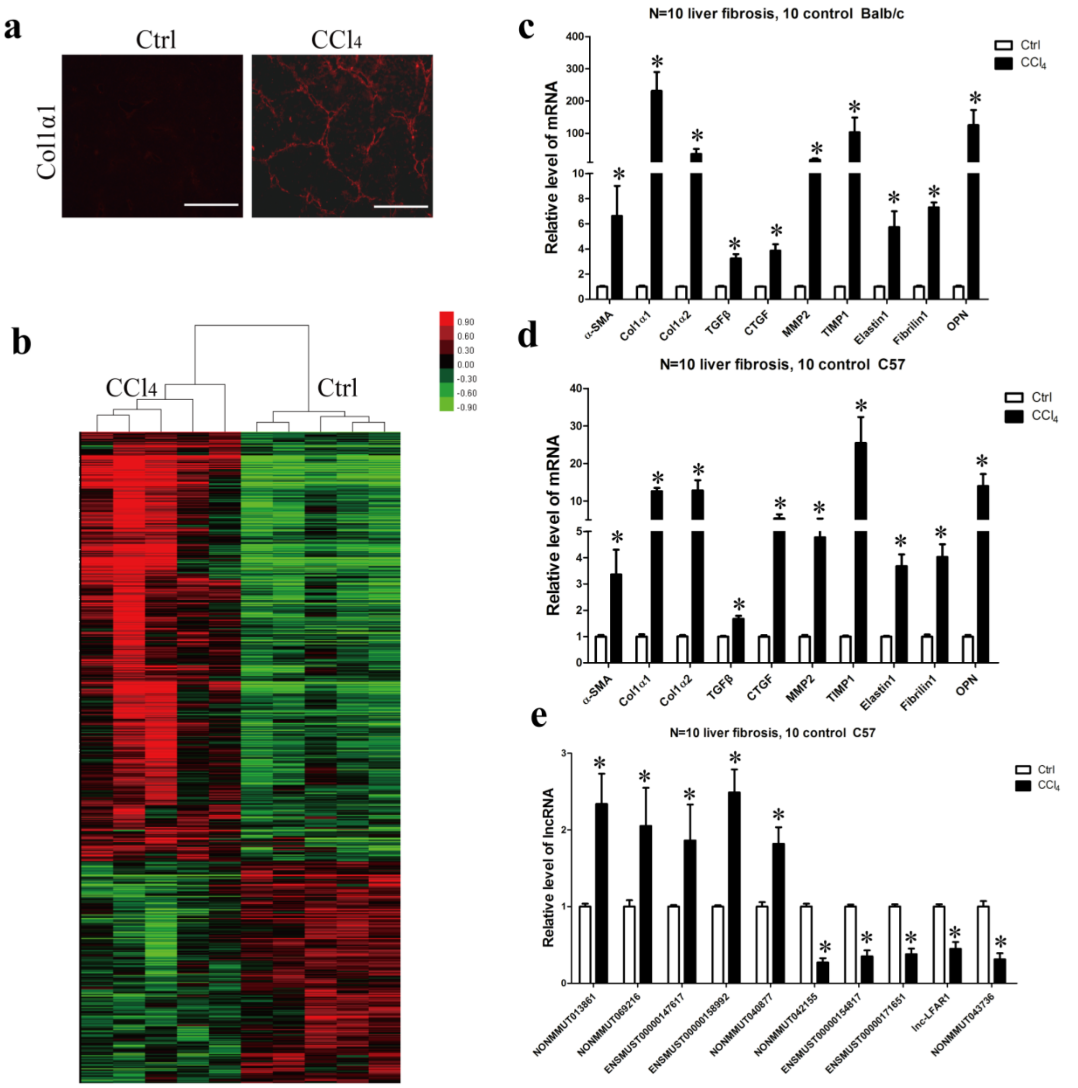

Supplementary Figure 1. Expression profiles of mRNAs on induction of liver fibrosis. Mice were injected six weeks with either olive oil or CCl_4 to induce liver fibrosis and were sacrificed two days after the last injection. **(a)** Liver tissues were harvested and stained using immunofluorescent assay for collagen1. Scale bars, 400 μm. **(b)** Microarray analysis for mRNA was performed with RNA extracts from extracts from livers of CCl_4-treated (n = 5) and oil-treated (n = 5) Balb/c mice (6 weeks' treatment). Hierarchical cluster analysis of significantly differentially expressed mRNAs: bright green, under-expression; gray, no change; bright red, over-expression. **(c)** Differential expression of ten representative mRNAs was validated in fibrotic and normal liver tissues by qRT-PCR in Balb/c mice (n = 10 per group). **(d)** Differential expression of ten representative mRNAs was validated in fibrotic and normal liver tissues by qRT-PCR in C57 mice (n = 10 per group). **(e)** Differential expression of ten

representative lncRNAs was validated in fibrotic and normal liver tissues by qRT-PCR in C57 mice (n = 10 per group). Data are presented as means ± s.e.m. *P* values were analysed by Student's *t*-test. **P*< 0.05.

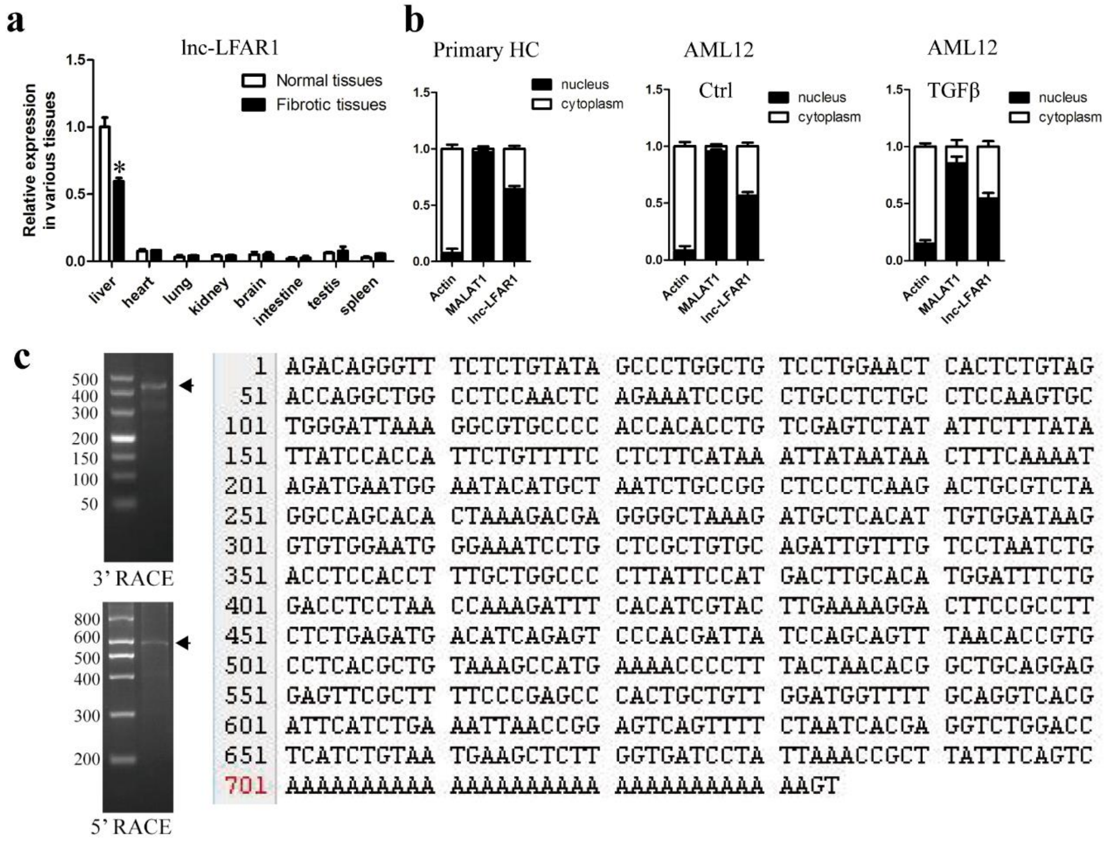

Supplementary Figure 2. Characteristics of the liver-enriched lnc-LFAR1. (a) qRT-PCR analysis of *lnc-LFAR1* in various tissues from normal and fibrotic Balb/c mice. **(b)** RNA was extracted from the nuclei or cytoplasm of primary HCs or AML12 cells treated with or without TGFβ. 1 μg of RNA was used for the qRT-PCR analysis of *lnc-LFAR1*, *lnc-MALAT1* (nuclear retained), and *β-actin* mRNAs (cytoplasm retained). **(c)** Agarose gelelectrophoresis of nested PCR products from the 5'-RACE procedure and 3'-RACE procedure. The molecular weight markers (base pairs) are indicated on the side. The major PCR product is marked with an arrow. Nucleotide sequence of the full-length lnc-LFAR1 was confirmed by RACE. The number of biological replicates for each experiment was n = 3. Data are presented as means ± s.e.m. *P* values were analysed by Student's *t*-test. **P* < 0.05.

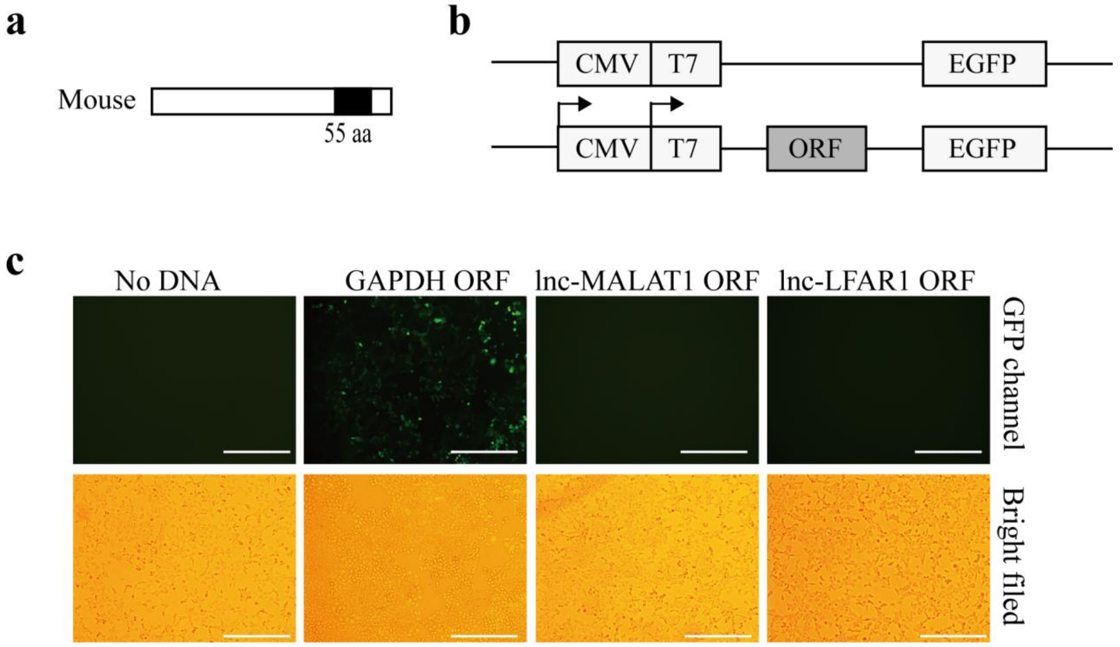

Supplementary Figure 3. Lnc-LFAR1 is a long non-coding RNA. (a) Predicted ORFs of lnc-LFAR1, the black box shows the predicted ORFs in mice. **(b, c)** GAPDH, lnc-LFAR1 (predicted ORF), and lnc-MALAT1 (predicted ORF) were cloned, respectively, into pcDNA3.1(+) with C-terminal EGFP-tag **(b)**. **(c)** IF analysis of the EGFP expression in AML12 cells transfected with the indicated constructs. Scale bars, 400 μm.

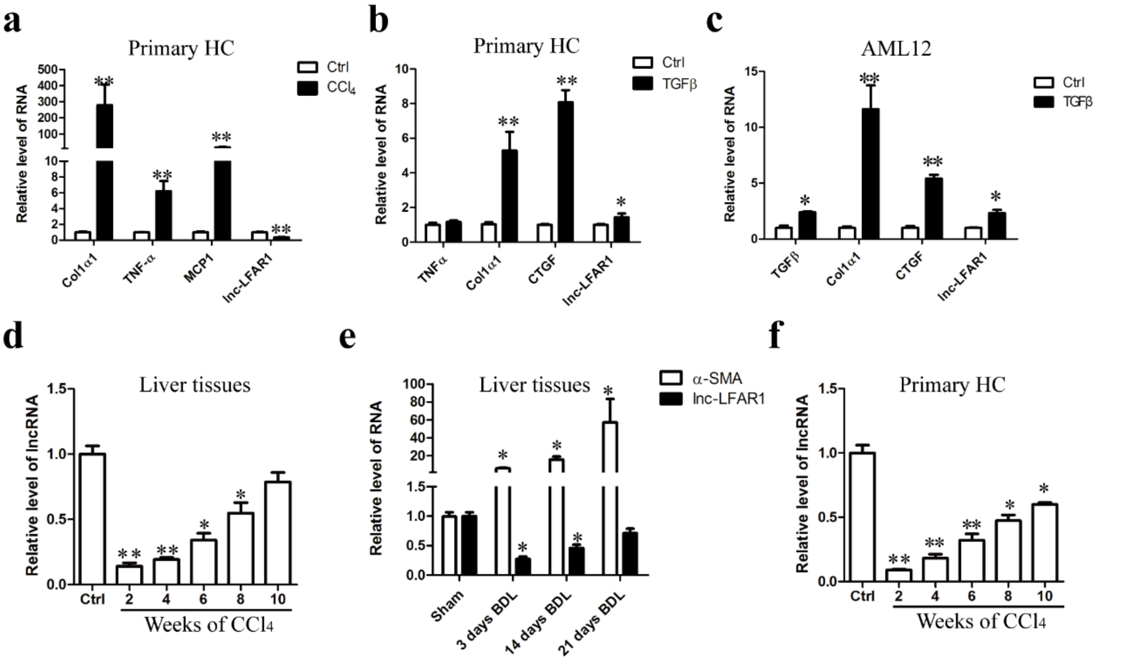

Supplementary Figure 4. The expression profiling of lnc-LFAR1. (a) Primary HCs were isolated from livers of Balb/c mice treated for 6 weeks with CCl_4 or oil, and the RNA levels of *lnc-LFAR1*, *Col1α1*, *TNFα* and *MCP1* were determined by qRT-PCR. **(b, c)** Primary HCs **(b)** and AML12 cells **(c)** were stimulated with TGFβ for 24 h and the expression of *lnc-LFAR1* and positive control genes (*Col1α1* and *CTGF*) was determined by qRT-PCR. **(d)** qRT-PCR analysis of the expression of *lnc-LFAR1* in livers from mice treated with CCl_4 for 2 (2w CCl_4), 4 (4w CCl_4), 6 (6w CCl_4), 8 (8w CCl_4) or 10 (10w CCl_4) weeks. **(e)** qRT-PCR analysis of the expression of *α-SMA* and *lnc-LFAR1* in livers from mice that underwent bile duct ligation for 3, 14 or 21 days. **(f)** qRT-PCR analysis of the expression of *lnc-LFAR1* in primary HCs isolated from mice treated with CCl_4 for 2 (2w CCl_4), 4 (4w CCl_4), 6 (6w CCl_4), 8 (8w CCl_4) or 10 (10w CCl_4) weeks. The number of biological replicates for each experiment was $n \geq 3$. Data are presented as means ± s.e.m. *P* values were analysed by Student's *t*-test. $^*P < 0.05$, $^{**}P < 0.01$.

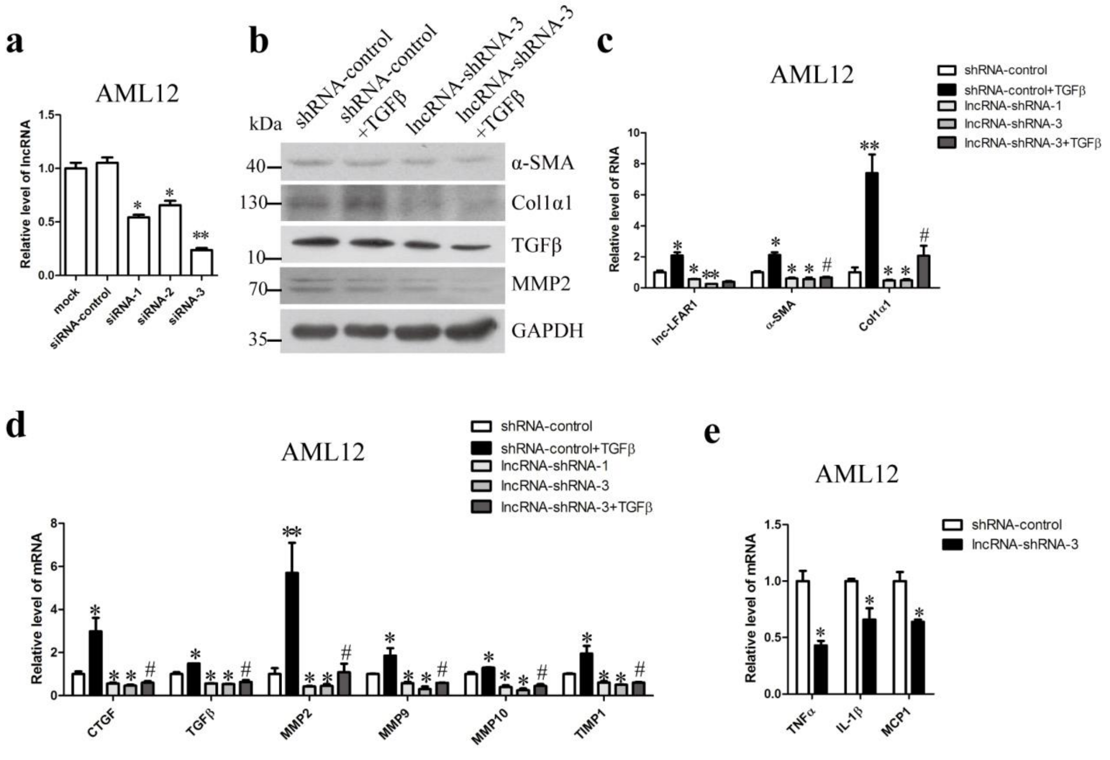

Supplementary Figure 5. Knockdown of lnc-LFAR1 reduces TGFβ-induced pro-fibrogenic gene expression in AML12 cells. (a) Targeting of lnc-LFAR1 in AML12 cells by three independent siRNAs led to significant depletion of the transcript as measured by qRT-PCR. **(b)** AML12 cells were infected with lentivirus-mediated shLFAR1 for 72 h and further treated with 10 ng ml^{-1} TGFβ for additional 24 h. The protein levels of α-SMA, Col1α1, TGFβ and MMP2 were detected by western blot. GAPDH was used as an internal control. Uncropped blots of this figure accompanied by the location of molecular weight markers are shown in Supplementary Fig.19. **(c-e)** AML12 cells were infected with lentivirus-mediated shLFAR1 (shRNA1 and shRNA3) for 72 h and further treated with 10 ng ml^{-1} TGFβ for additional 24 h. The expression of *lnc-LFAR1, α-SMA, Col1α1* **(c)**, *TGFβ, CTGF, TGFβR1, MMP2/9/10, TIMP1* **(d)**, *TNFα, IL-1β* and *MCP1* **(e)** was detected by qRT-PCR. In **a, c, d** and **e**, the number of biological replicates for each experiment was $n \geq 3$. Data are presented as means ± s.e.m. *P* values were analysed by one-way ANOVA followed by *post hoc* comparison in **c** and **d**, and by Student's *t*-test in **a** and **e**. */#P<0.05, **P<0.01. *P<0.05 vs mock in **a**; *P<0.05 vs shRNA-control, #P<0.05 vs shRNA-control+TGFβ in **c**, **d** and **e**.

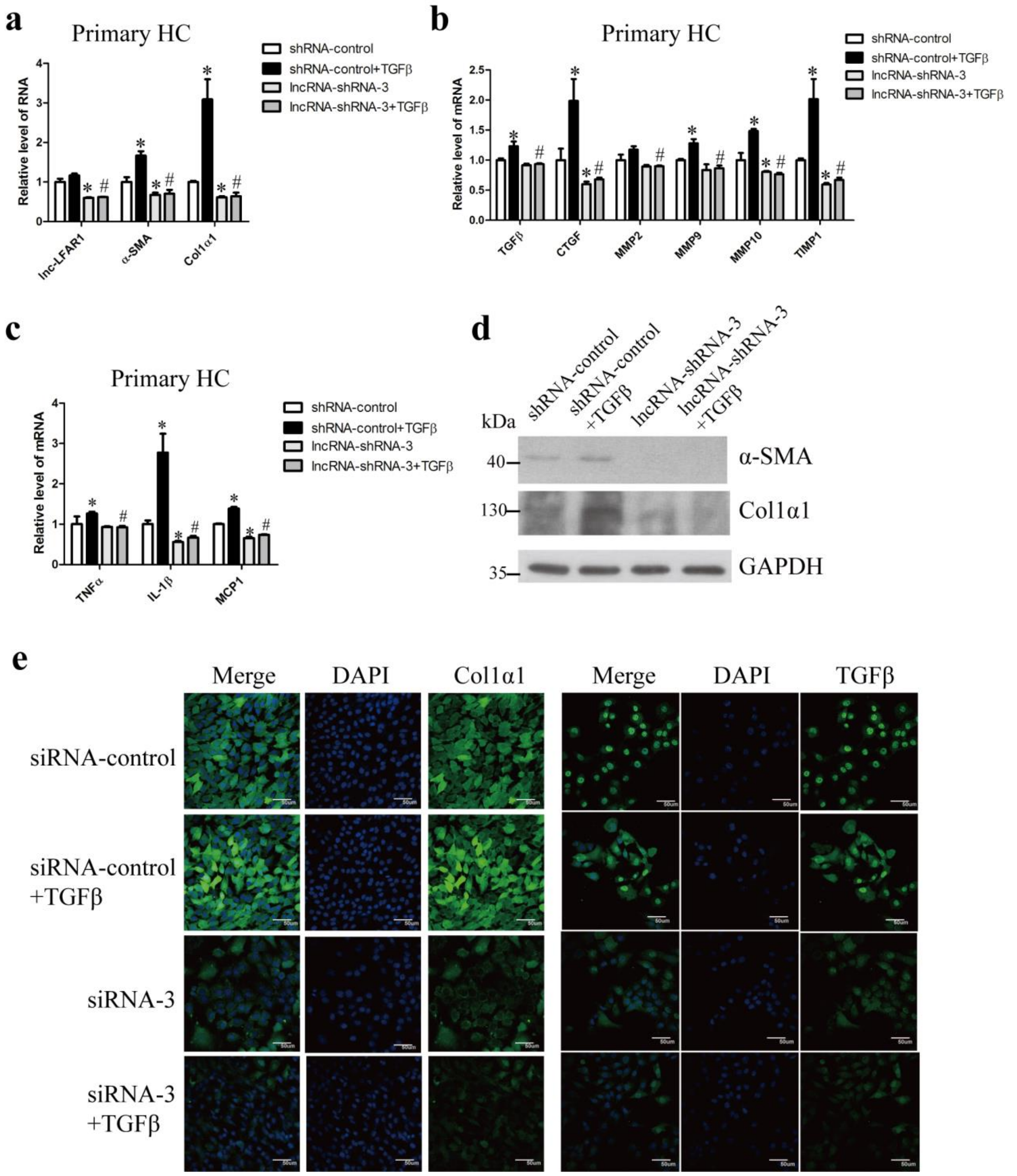

Supplementary Figure 6. Knockdown of lnc-LFAR1 reduces TGFβ-induced pro-fibrogenic gene expression in hepatocytes. **(a-d)** Primary HCs were infected with lentivirus-mediated shLFAR1 for 72 h and further treated with TGFβ for additional 24h. The expression of pro-fibrogenic genes **(a-b)** and pro-inflammation genes **(c)** was detected by qRT-PCR **(a-c)** and western blot **(d)**. Uncropped blots of this figure accompanied by the location of molecular weight markers are shown in Supplementary Fig.19. **(e)** AML12 cells were transfected with siRNA for lnc-LFAR1 for 48 h and further treated with TGFβ for additional 24 h. The expression of Col1α1 and TGFβ were determined by confocal microscopy. DAPI stained nuclei blue; scale bar, 50 μm. In **a**, **b** and **c**, the number of biological replicates for each experiment was $n \geq 3$. Data are presented as means ± s.e.m. *P* values were analysed

by one-way ANOVA followed by *post hoc* comparison. */# $P<0.05$. * $P<0.05$ vs shRNA-control, # $P<0.05$ vs shRNA-control+TGFβ.

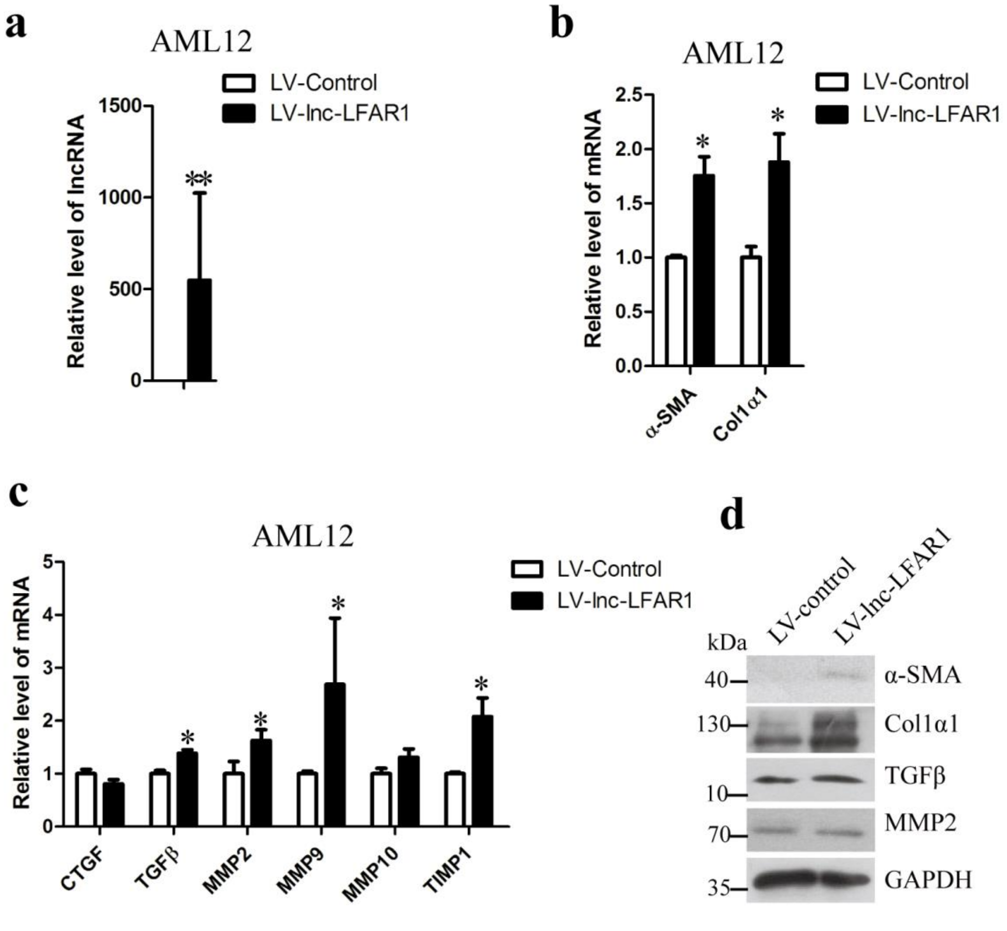

Supplementary Figure 7. Lnc-LFAR1 promotes pro-fibrogenic genes expression in AML12 cells. **(a)** Relative lncRNA expression level of *lnc-LFAR1* in AML12 cells infected with lenti-lnc-LFAR1 or lenti-control was examined by qRT-PCR. **(b-d)** The expression of pro-fibrogenic genes was detected in lnc-LFAR1 up-regulated AML12 cells by qRT-PCR **(b, c)** and western blot **(d)**. Uncropped blots of this figure accompanied by the location of molecular weight markers are shown in Supplementary Fig.19. The number of biological replicates for each experiment was $n \geq 3$. Data are presented as means ± s.e.m. P values were analysed by Student's t-test. $^{*}P < 0.05$, $^{**}P < 0.01$.

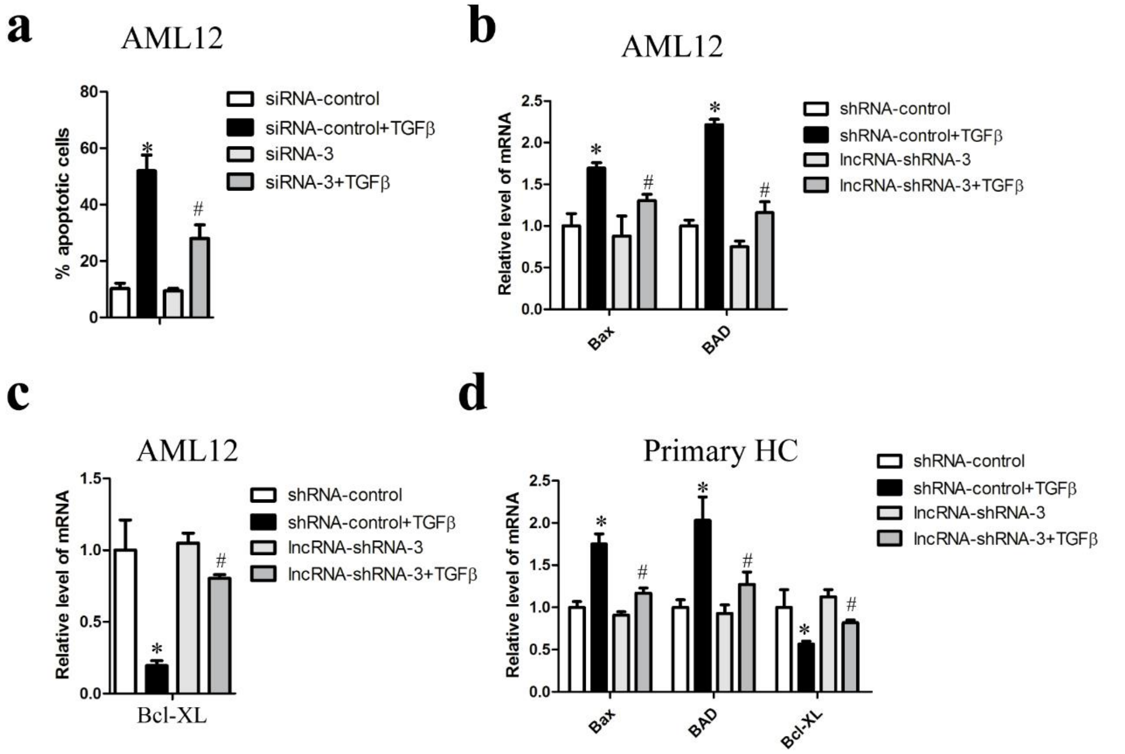

Supplementary Figure 8. Knockdown of lnc-LFAR1 reduces TGFβ-induced apoptosis in hepatocytes. (a) AML12 cells were transfected with siRNA for lnc-LFAR1 or siRNA-control for 48h and then treated with or without TGFβ for additional 48 h. Cell apoptosis were determined by FACS analysis. **(b-d)** The expression of *Bax, Bad* and *Bcl-XL* was detected in lnc-LFAR1 down-regulated AML12 cells **(b, c)** and primary HCs **(d)** treated with or without TGFβ by qRT-PCR. The number of biological replicates for each experiment was n ≥ 3. Data are presented as means ± s.e.m. *P* values were analysed by one-way ANOVA followed by *post hoc* comparison. */#P<0.05. *P<0.05 vs shRNA-control, #P<0.05 vs shRNA-control+TGFβ.

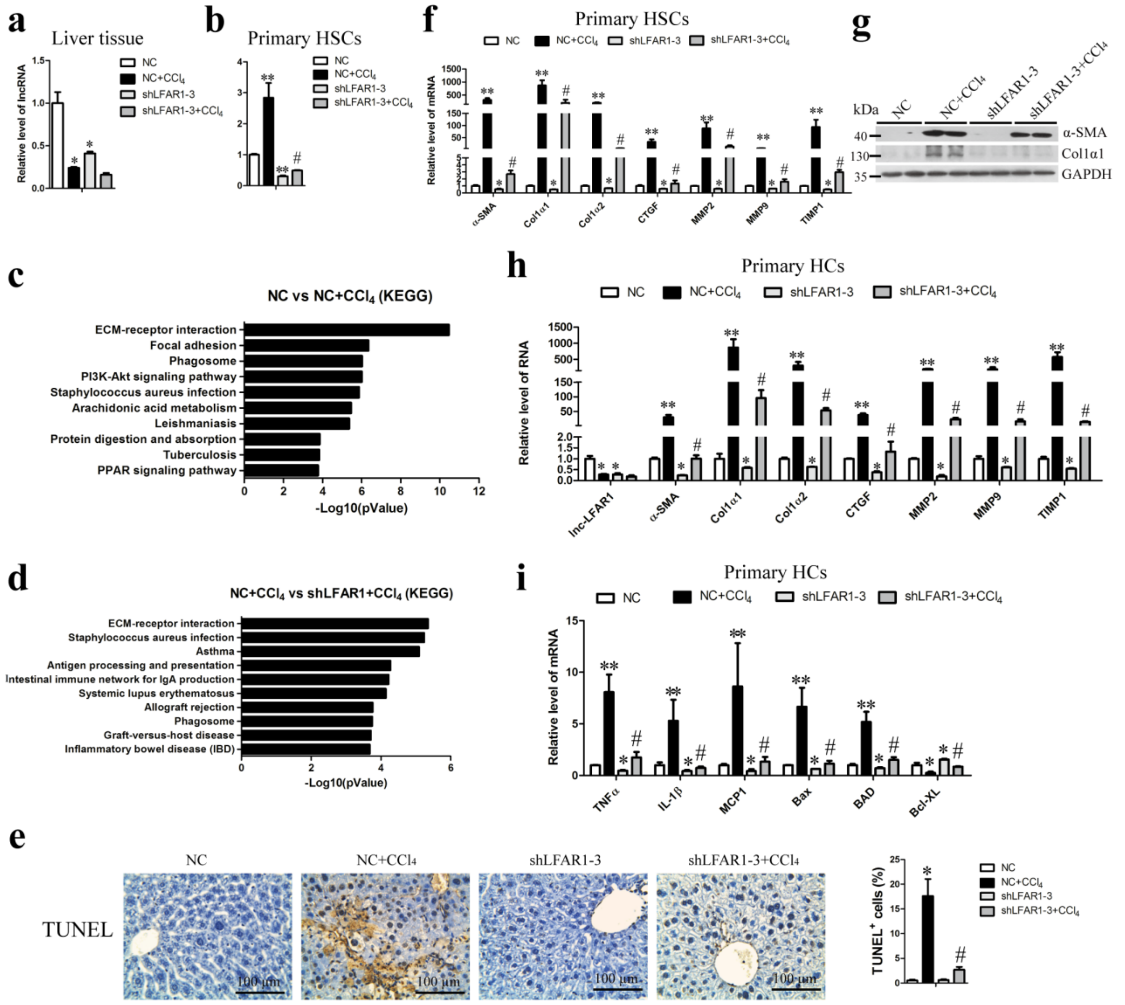

Supplementary Figure 9. Knockdown of lnc-LFAR1 attenuates CCl_4-induced liver fibrosis *in vivo*.

Mice were treated with oil in combination with injection of lenti-NC (NC, n = 10), or CCl_4 in combination with injection of lenti-NC (NC+CCl_4, n = 10), or oil in combination with injection of lenti-shLFAR1 (shLFAR1, n = 10), or CCl_4 in combination with injection of lenti-shLFAR1 (shLFAR1+CCl_4, n = 10). **(a)** Relative lncRNA expression level of *lnc-LFAR1* in each group was examined by qRT-PCR (n = 5 per group). **(b)** qRT-PCR analysis of lnc-LFAR1 level in the HSCs, which were isolated from mice in each group (n = 3 per group). (**c, d**) KEGG pathway analysis was performed between the four groups. **(f)** qRT-PCR analysis of pro-fibrogenic genes levels in the HSCs, which were isolated from mice in each group (n = 3 per group). **(g)** Western blot analysis for α-SMA and Col1α1 protein levels in HSCs that isolated from mice in each group. Uncropped blots of this figure accompanied by the location of molecular weight markers are shown in Supplementary Fig. 19. **(h, i)** qRT-PCR analysis of lnc-LFAR1, pro-fibrogenic genes, pro-inflammation genes and apoptosis-related genes levels in the HCs, which were isolated from mice in each group (n = 3 per

group). **(e)** Apoptosis levels was detected by TUNEL staining. Scale bars, 100 μm. Right, five images of each liver and five livers from different mice were quantified for each group. Data are presented as means ± s.e.m. *P* values were analysed by one-way ANOVA followed by *post hoc* comparison. */#$P<0.05$, **/##$P<0.01$. *$P<0.05$ vs NC, #$P<0.05$ vs NC+ CCl_4.

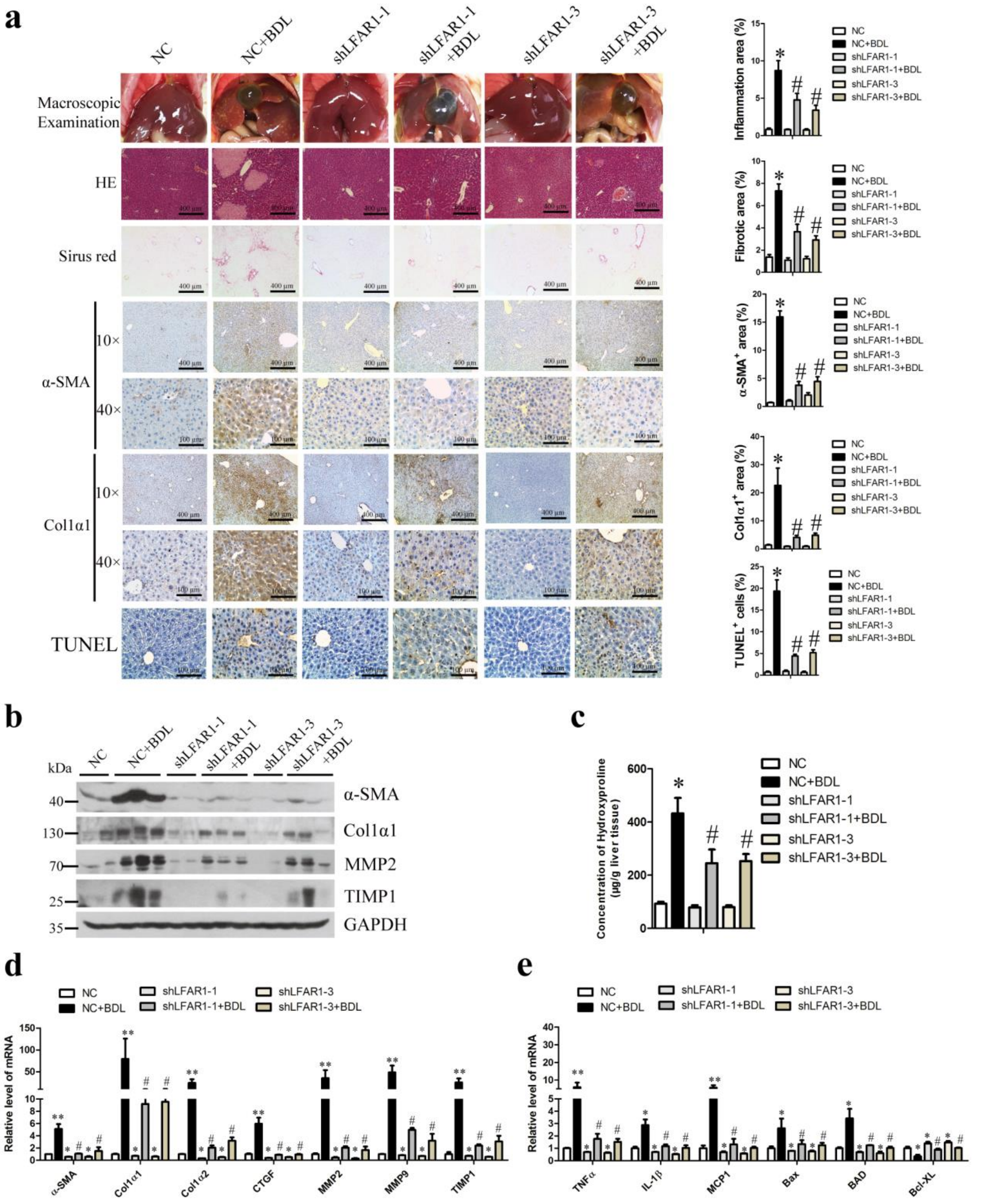

Supplementary Figure 10. Knockdown of lnc-LFAR1 attenuates BDL-induced liver fibrosis *in vivo*. Mice were treated with sham operation in combination with injection of lenti-NC (NC, n = 15), BDL operation in combination with injection of lenti-NC (NC+BDL, n = 15), sham operation in combination with injection of lenti-shLFAR1-1 (shLFAR1-1, n = 15), BDL operation in combination with injection of lenti-shLFAR1-1 (shLFAR1-1+BDL, n = 15), sham operation in combination with injection of lenti-shLFAR1-3 (shLFAR1-3, n = 15) and BDL operation in combination with injection of lenti-shLFAR1-1 (shLFAR1-3+BDL, n = 15). **(a)** Liver fibrosis was evaluated by macroscopic examination, H&E staining, Sirius red staining, IHC for α-SMA and collagen1 and TUNEL staining.

Scale bars, 400 μm for H&E staining, Sirius red staining and IHC (objective, ×10); 100 μm for IHC (objective, ×40) and TUNEL staining. Right, five images of each liver and five livers from different mice were quantified for each group. **(b)** The protein levels of α-SMA, Col1α1, MMP2 and TIMP1 were determined by western blot. GAPDH was used as an internal control. Uncropped blots of this figure accompanied by the location of molecular weight markers are shown in Supplementary Fig. 19. **(c)** Quantification of hepatic hydroxyproline content. The data are expressed as hydroxyproline (μg)/liver wet weight (g) (n = 8 per group). **(d, e)** The mRNA levels of hepatic pro-fibrogenic genes (*α-SMA, Col1α1, Col1α2, CTGF, MMP2/9* and *TIMP1*) **(d)**, pro-inflammation genes (*TNFα, IL1β* and *MCP1*) and apoptosis-related genes (*Bax, BAD* and *Bcl-XL*) **(e)** were determined by qRT-PCR. In **d** and **e**, the number of biological replicates for each experiment was n ≥ 3. Data are presented as means ± s.e.m. *P* values were analysed by one-way ANOVA followed by *post hoc* comparison in **a**, **c-e**. */#*P*<0.05, **/##*P*<0.01. **P*<0.05 vs NC, #*P*<0.05 vs NC+ BDL.

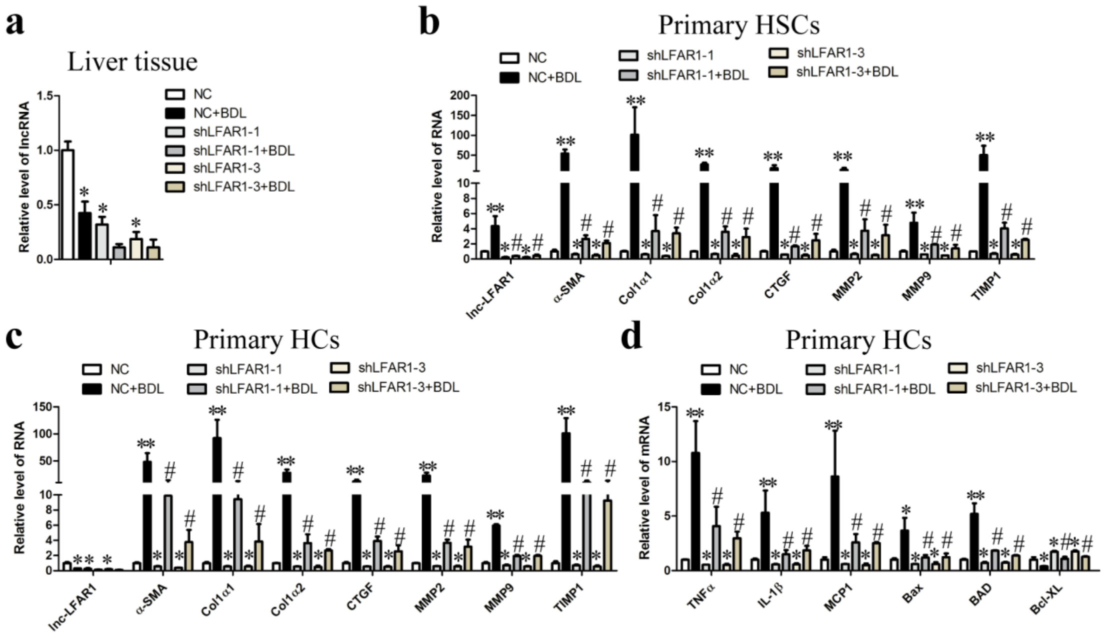

Supplementary Figure 11. Knockdown of lnc-LFAR1 attenuates BDL-induced liver fibrosis *in vivo*. Mice were treated with sham operation in combination with injection of lenti-NC (NC, n = 15), BDL operation in combination with injection of lenti-NC (NC+BDL, n = 15), sham operation in combination with injection of lenti-shLFAR1-1 (shLFAR1-1, n = 15),BDL operation in combination with injection of lenti-shLFAR1-1 (shLFAR1-1+BDL, n = 15), sham operation in combination with injection of lenti-shLFAR1-3 (shLFAR1-3, n = 15) and BDL operation in combination with injection of lenti-shLFAR1-1 (shLFAR1-3+BDL, n = 15). **(a)** Relative lncRNA expression level of *lnc-LFAR1* in each group was examined by qRT-PCR (n = 5 per group). **(b)** qRT-PCR analysis of *lnc-LFAR1* and pro-fibrogenic genes levels in the HSCs, which were isolated from mice in each group (n = 3 per group). (**c, d**) qRT-PCR analysis of *lnc-LFAR1*, pro-fibrogenic genes, pro-inflammation genes and apoptosis-related genes levels in the HCs, which were isolated from mice in each group (n = 3 per group). Data are presented as means ± s.e.m. *P* values were analysed by one-way ANOVA followed by *post hoc* comparison. */#*P*<0.05, **/##*P*<0.01. **P*<0.05 vs NC, #*P*<0.05 vs NC+ BDL.

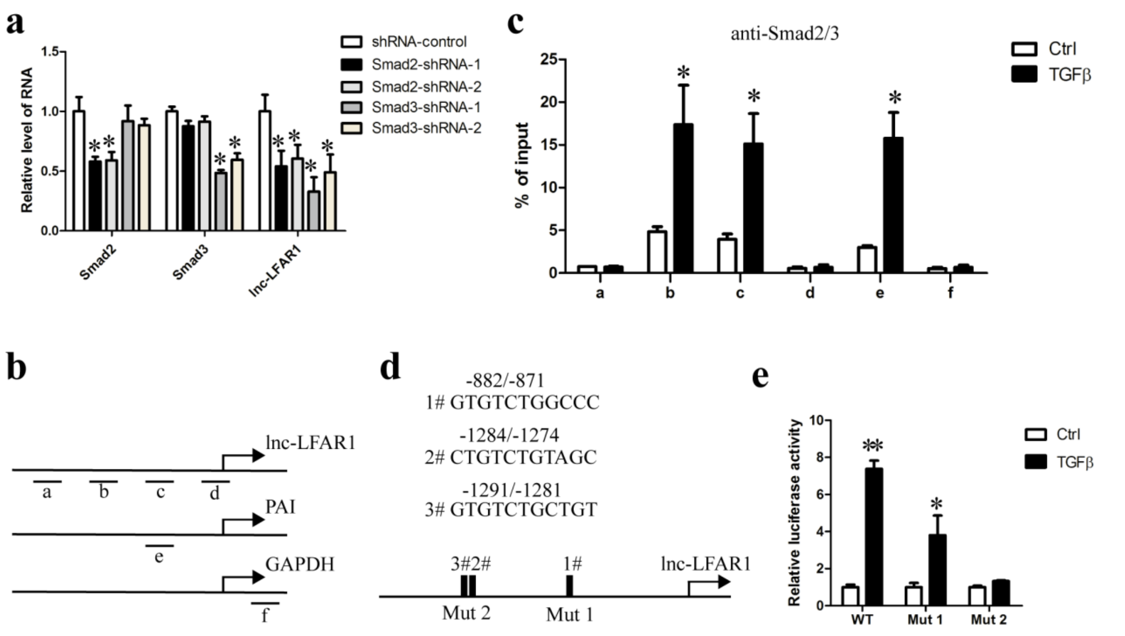

Supplementary Figure 12. Smad2/3 mediates TGFβ-induced lnc-LFAR1 expression in AML12 cells. (a) Relative expression levels of *Smad2*, *Smad3* and *lnc-LFAR1* in AML12 cells infected with lenti-shSMAD2 or lenti-shSMAD3 or lenti-control virus were examined by qRT-PCR. **(b, c)** ChIP analyses of AML12 cells treated with or without 10 ng ml^{-1} TGFβ for 24 h were conducted on lnc-LFAR1 (primer set a-d), PAI (the positive control; primer e) and GAPDH (the negative control; primer f) promoter regions using anti-Smad2/3 antibody. Enrichment was shown relative to input. **(d)** Diagram of the predicted three Smad2/3 binding sites in the lnc-LFAR1 promoter region. Points mutation of binding site 1 (Mut1), and binding sites 2 and 3 (Mut2) of Smad2/3 were indicated. **(e)** Luciferase analysis. AML12 cells were transfected with the luciferase reporter constructs harboring either Smad2/3 binding sites or the mutated binding sites for 48 h, and further treated with 10 ng ml^{-1} TGFβ for additional 24 h. The cells were lysed for dual luciferase analysis. The renilla was transfected as an internal control. In **a, c** and **e**, the number of biological replicates for each experiment was $n \geq 3$. Data are presented as means ± s.e.m. *P* values were analysed by Student's *t*-test. *P<0.05. *P<0.05 vs shRNA-control in **a**; and *P<0.05 vs Ctrl in **c** and **e**.

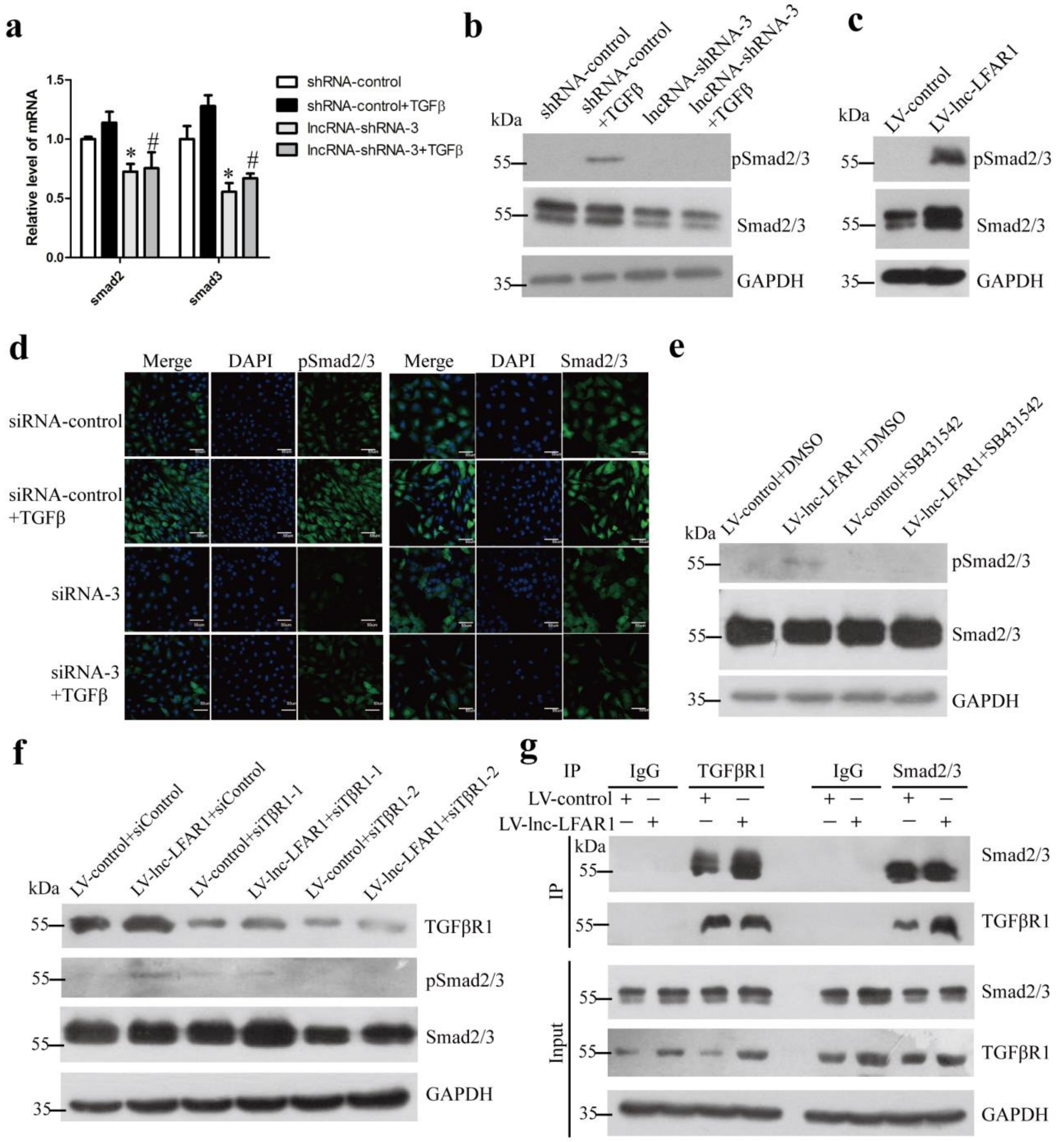

Supplementary Figure 13. Knockdown of lnc-LFAR1 dramatically decreases TGFβ-induced Smad2/3 phosphorylation and translocation in AML12 cells. (a, b) AML12 cells were infected with lentivirus-mediated shLFAR1 for 72 h and further treated with 10 ng ml^{-1} TGFβ for additional 24 h. *Smad2* and *Smad3* mRNA levels were determined by qRT-PCR **(a)**. pSmad2/3 and total Smad2/3 levels were detected by western blot **(b)**. **(c)** pSmad2/3 and total Smad2/3 levels were detected in lnc-LFAR1 up-regulated AML12 cells by western blot. GAPDH was used as an internal control. **(d)** AML12 cells were transfected with siRNA for lnc-LFAR1 for 48 h and further treated with 10 ng ml^{-1} TGFβ for additional 24 h. The expression and location of pSmad2/3 and total Smad2/3 were determined by confocal microscopy. DAPI stained nuclei blue; scale bar, 50μm. **(e)** AML12 cells were infected with LV-lnc-LFAR1 for 72 h and further treated with TGFβR1 inhibitor SB431542 for additional 48 h.

pSmad2/3 and total Smad2/3 levels were detected by western blot. GAPDH was used as an internal control. **(f)** AML12 cells were infected with LV-lnc-LFAR1 for 72 h and further transfected with siRNA for TGFβR1 for additional 48 h. TGFβR1, pSmad2/3 and total Smad2/3 levels were detected by western blot. GAPDH was used as an internal control. **(g)** TGFβR1 and Smad2/3 antibodies were used for co-immunoprecipitation (IP) with AML12 cell lysates infected with or without LV-lnc-LFAR1. Uncropped blots of this figure accompanied by the location of molecular weight markers are shown in Supplementary Fig. 19. In **a**, the number of biological replicates for each experiment was n ≥ 3. Data are presented as means ± s.e.m. *P* values were analysed by one-way ANOVA followed by *post hoc* comparison. */#*P*<0.05. **P*<0.05 vs shRNA-control, #*P*<0.05 vs shRNA-control+TGFβ.

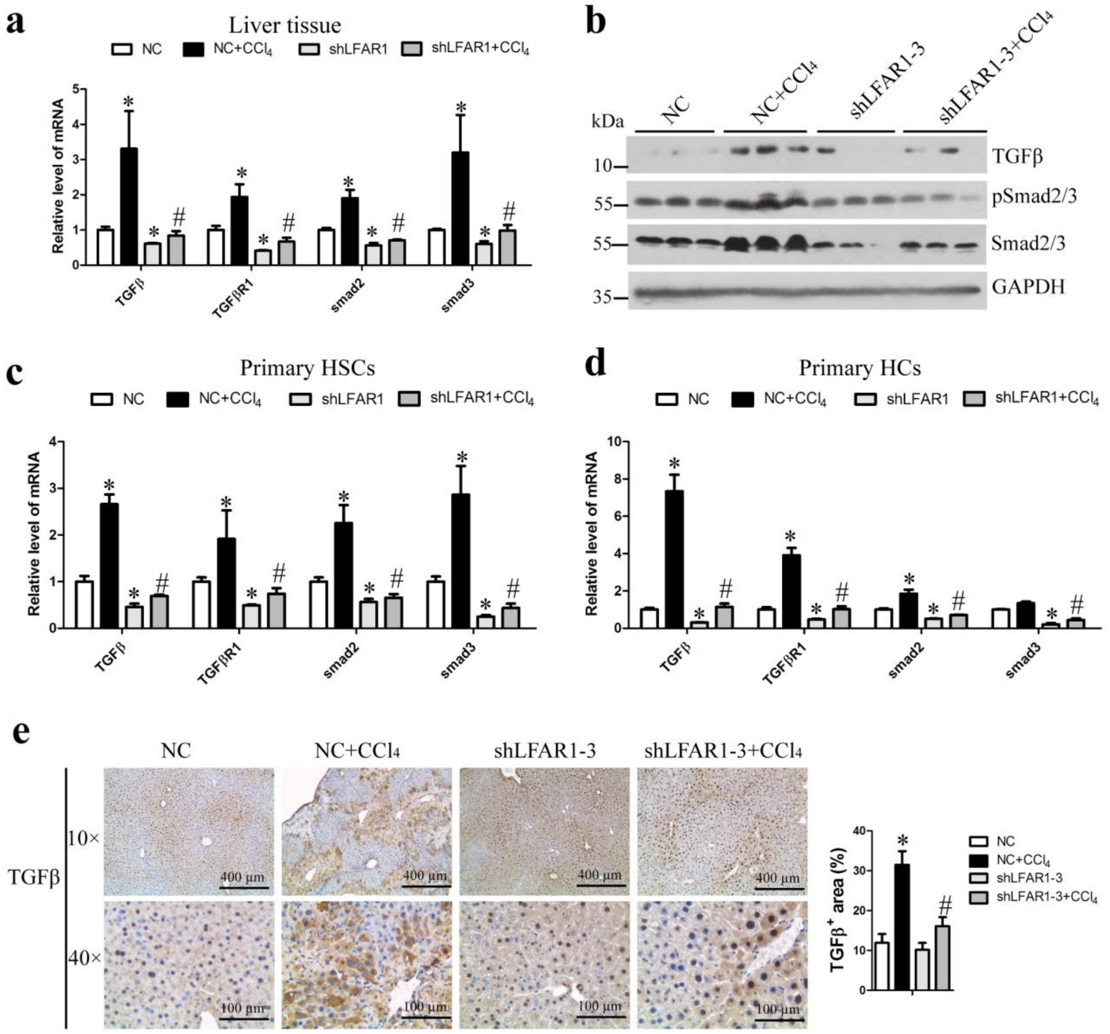

Supplementary Figure 14. Lnc-LFAR1 regulates the transcription of TGFβ, TGFβR1, Smad2 and Smad3. Mice were treated with oil in combination with injection of lenti-NC (NC, n = 10), or CCl_4 in combination with injection of lenti-NC (NC+CCl_4, n = 10), or oil in combination with injection of lenti-shLFAR1 (shLFAR1, n = 10), or CCl_4 in combination with injection of lenti-shLFAR1 (shLFAR1+CCl_4, n = 10). **(a)** *TGFβ*, *TGFβR1*, *Smad2* and *Smad3* levels were determined by qRT-PCR (n = 5). **(b)** TGFβ, pSmad2/3 and total Smad2/3 levels were detected in each group by western blot. Uncropped blots of this figure accompanied by the location of molecular weight markers are shown in Supplementary Fig. 19. **(c, d)** qRT-PCR analysis of *TGFβ*, *TGFβR1*, *Smad2* and *Smad3* levels in HSCs **(c)** and HCs **(d)**, which were isolated from mice in each group respectively (n = 3 per group). **(e)** TGFβ level was detected in each group by IHC. Scale bars, 400 μm for IHC (objective, ×10); 100 μm for IHC (objective, ×40). Right, five images of each liver and five livers from different mice were quantified for each group. Data are presented as means ± s.e.m. *P* values were analysed by one-way ANOVA followed by *post hoc* comparison. */#P<0.05. *P<0.05 vs NC, #P<0.05 vs NC+ CCl_4.

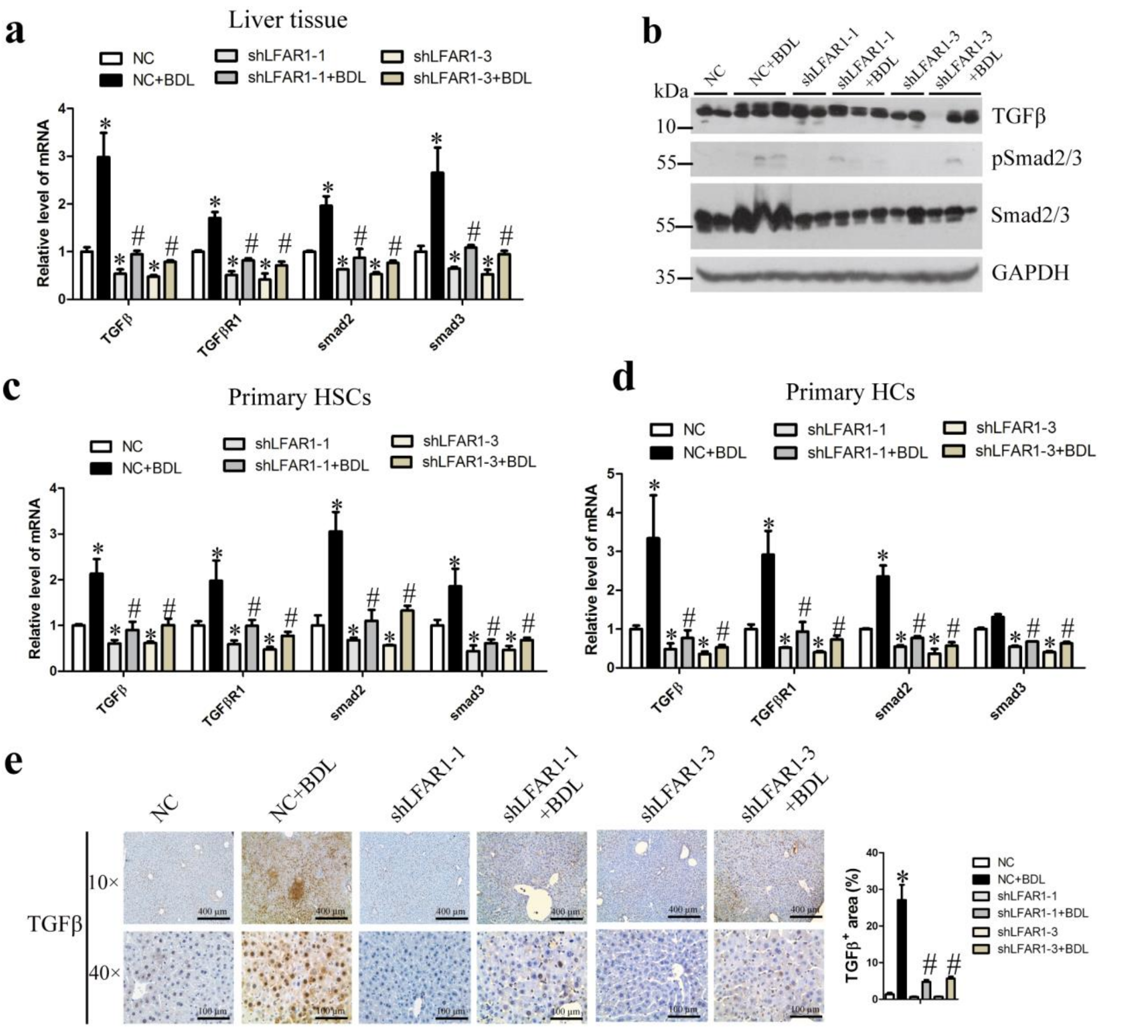

Supplementary Figure 15. Lnc-LFAR1 regulates the transcription of TGFβ, TGFβR1, Smad2 and Smad3. Mice were treated with sham operation in combination with injection of lenti-NC (NC, n = 15), BDL operation in combination with injection of lenti-NC (NC+BDL, n = 15), sham operation in combination with injection of lenti-shLFAR1-1 (shLFAR1-1, n = 15), BDL operation in combination with injection of lenti-shLFAR1-1 (shLFAR1-1+BDL, n = 15), sham operation in combination with injection of lenti-shLFAR1-3 (shLFAR1-3, n = 15) and BDL operation in combination with injection of lenti-shLFAR1-1 (shLFAR1-3+BDL, n = 15). **(a)** *TGFβ*, *TGFβR1*, *Smad2* and *Smad3* levels were determined by qRT-PCR (n = 5 per group). **(b)** TGFβ, pSmad2/3 and total Smad2/3 levels were detected in each group by western blot. GAPDH was used as an internal control. Uncropped blots of this figure accompanied by the location of molecular weight markers are shown in Supplementary Fig. 19. **(c, d)** qRT-PCR analysis of *TGFβ*, *TGFβR1*, *Smad2* and *Smad3* levels in HSCs **(c)** and HCs **(d)**, which were isolated from mice in each group respectively (n = 3 per group). **(e)** TGFβ level was detected in each group by IHC. Scale bars, 400 μm for IHC (objective, ×10); 100 μm for IHC

(objective, ×40). Right, five images of each liver and five livers from different mice were quantified for each group. Data are presented as means ± s.e.m. *P* values were analysed by one-way ANOVA followed by *post hoc* comparison. */#$P<0.05$. *$P<0.05$ vs NC, #$P<0.05$ vs NC+ BDL.

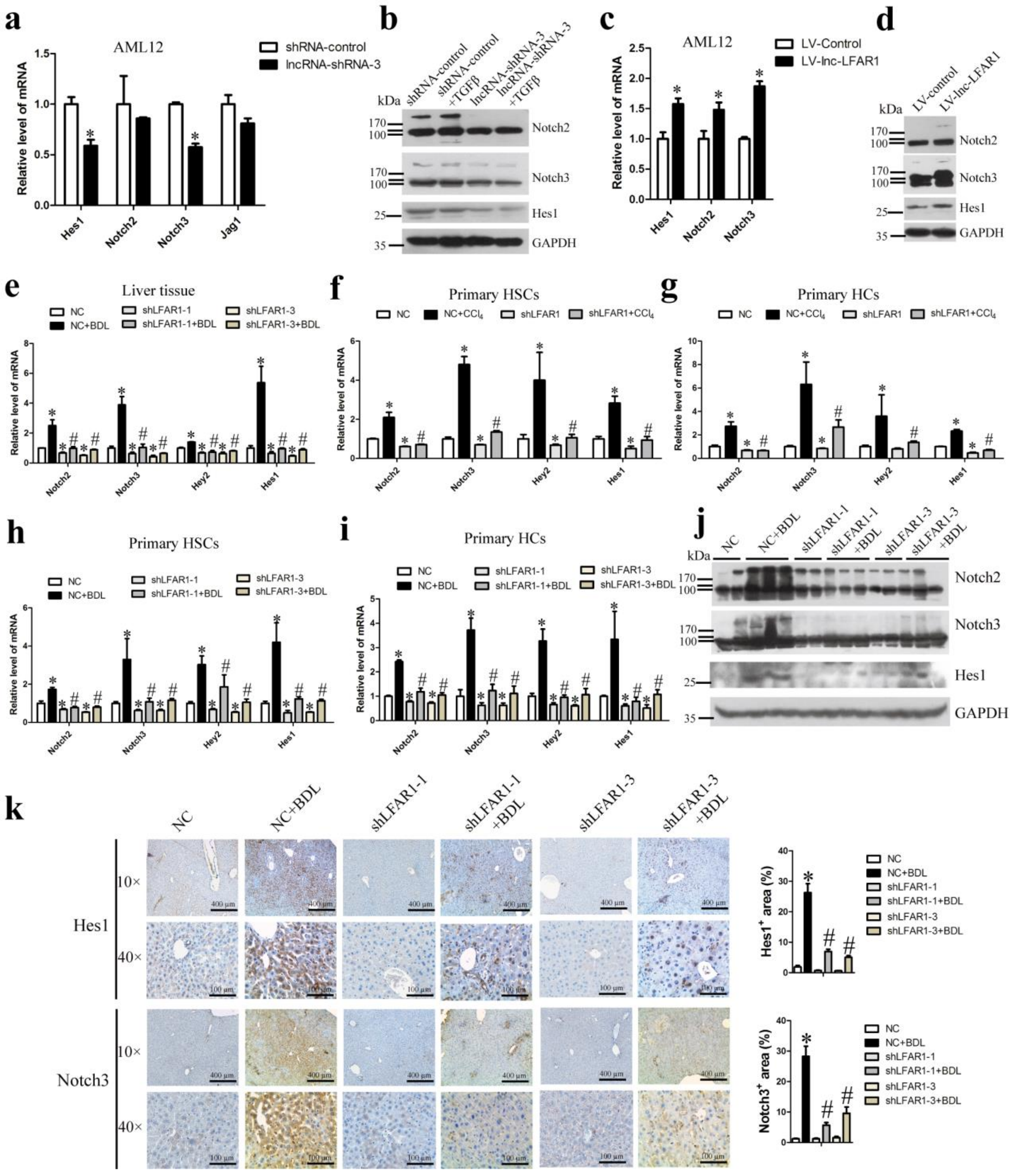

Supplementary Figure 16. Lnc-LFAR1 promotes Notch pathway activation. (a) The mRNA levels of *Hes1*, *Notch2*, *Notch3* and *Jag1* were detected in lnc-LFAR1 down-regulated AML12 cells. **(b)** AML12 cells were infected with lentivirus-mediated shLFAR1 and further treated with TGFβ for additional 24h. The protein levels of Nocth2, Notch3 and Hes1 levels were detected by western blot. **(c, d)** The expression of *Nocth2*, *Notch3* and *Hes1* was detected in lnc-LFAR1 up-regulated AML12 cells by qRT-PCR **(c)** and western blot **(d)**. **(f, g)** Mice were treated with lenti-NC (n = 10), or NC+CCl_4 (n = 10), or shLFAR1 (n = 10), or shLFAR1+CCl_4 (n = 10). The mRNA levels of *Hes1*, *Notch2*, *Notch3* and *Hey2* were determined in the HSCs **(f)** and HCs **(g)**, which were isolated from mice in each group, respectively, by qRT-PCR. **(e, h-k)** Mice were treated with lenti-NC (n = 15), NC+BDL (n = 15),

shLFAR1-1 (n = 15), shLFAR1-1+BDL (n = 15), shLFAR1-3 (n = 15) and shLFAR1-3+BDL (n = 15). The mRNA levels of *Hes1*, *Notch2*, *Notch3* and *Hey2* were determined in liver tissues **(e)**, HSCs **(f)** and HCs **(g)**, which were isolated from mice in each group respectively, by qRT-PCR. The protein levels of Hes1, Notch2, Notch3 and Hey2 were determined in liver tissues **(j)**. Notch3 and Hes1 levels were detected by IHC. Scale bars, 400 µm for IHC (objective, ×10); 100 µm for IHC (objective, ×40). Right, five images of each liver and five livers from different mice were quantified for each group **(k)**. Uncropped blots of this figure accompanied by the location of molecular weight markers are shown in Supplementary Fig. 19. In **a**, **c** and **e-i**, the number of biological replicates for each experiment was n ≥ 3. Data are presented as means ± s.e.m. *P* values were analysed by Student's *t*-test in **a** and **c,** and by one-way ANOVA followed by *post hoc* comparison in **e-i** and **k**. */#*P*<0.05. **P*<0.05 vs shRNA-control in **a**; **P*<0.05 vs LV-Control in **c**;**P*<0.05 vs NC, #*P*<0.05 vs NC+ CCl_4 in **f** and **g**; **P*<0.05 vs NC, #*P*<0.05 vs NC+ BDL in **e**, **h**, **i** and **k**.

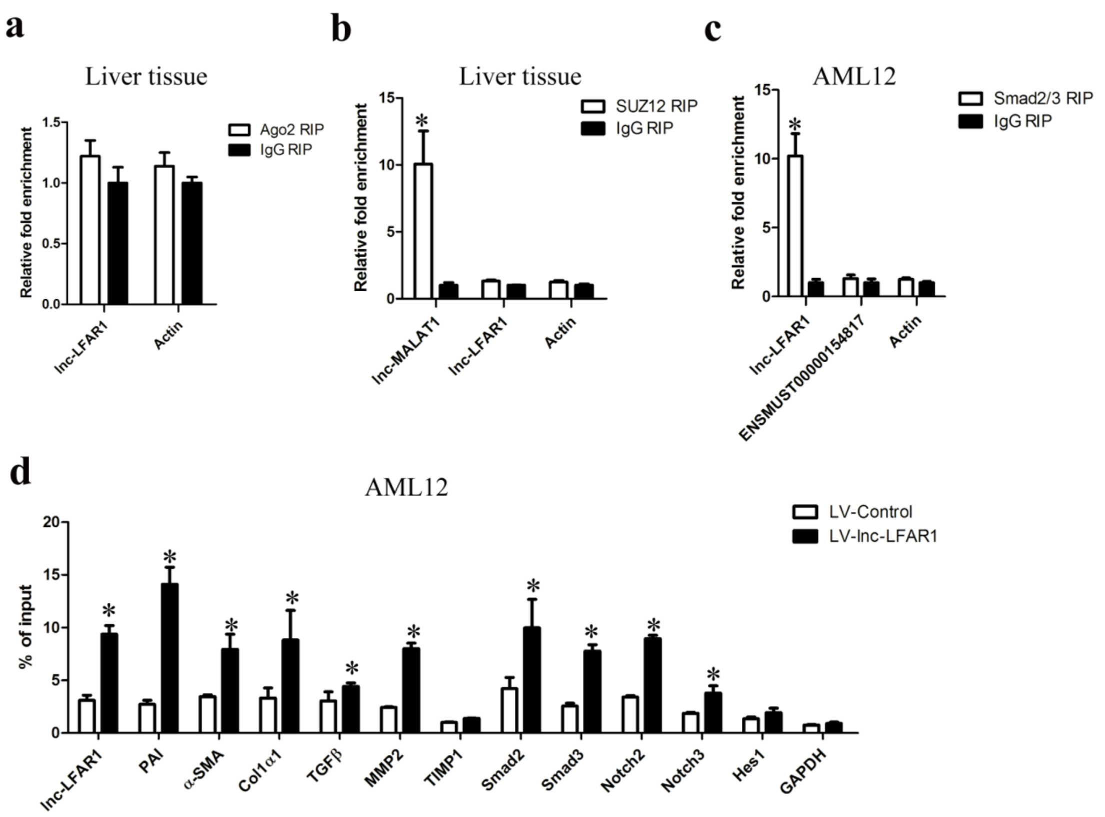

Supplementary Figure 17. Lnc-LFAR1 interacts with Smad2/3 in AML12 cells. (a) qRT-PCR detection of *lnc-LFAR1* retrieved by Ago2-specific antibody compared with IgG in the RIP assay within the single cell suspensions isolated from mouse liver. **(b)** qRT-PCR detection of *lnc-LFAR1* and *lnc-MALAT1* (positive control) retrieved by SUZ12-specific antibody compared with IgG in the RIP assay within the single cell suspensions isolated from mouse liver. **(c)** qRT-PCR detection of *lnc-LFAR1*, *lncRNA-ENSMUST00000154817* and *Actin* retrieved by Smad2/3-specific antibody compared with IgG in the RIP assay within AML12 cells infecting with lenti-LFAR1. **(c)** AML12 cells were infected with lenti-lnc-LFAR1 or lenti-control, and ChIP analyses were performed on indicated genes promoter regions using anti-Smad2/3 antibody. Enrichment was shown relative to input. The number of biological replicates for each experiment was n $\geq$ 3. Data are presented as means $\pm$ s.e.m. *P* values were analysed by Student's *t*-test. **P*<0.05. **P*<0.05 vs IgG RIP in **a-c**; and **P*<0.05 vs LV-Control in **d**.

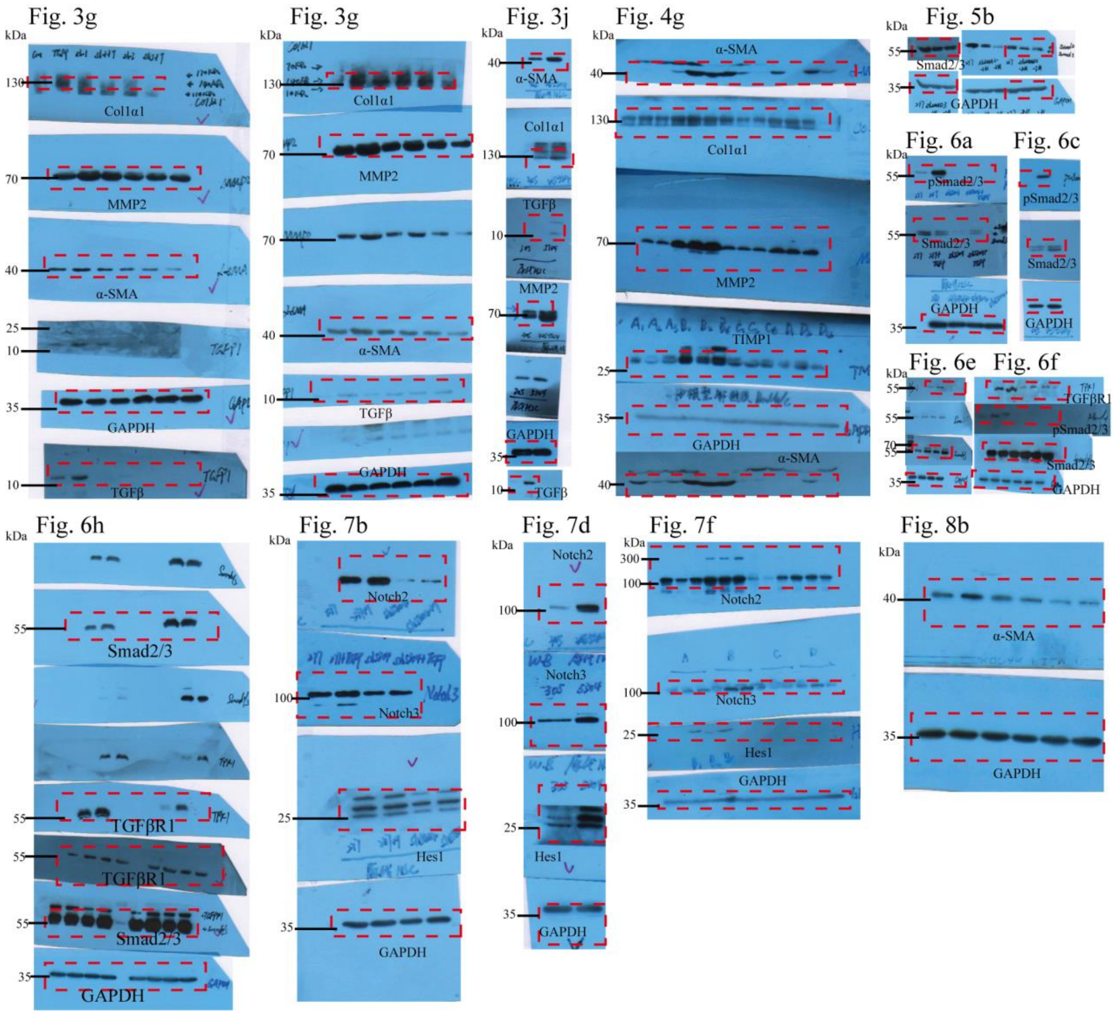

Supplementary Figure 18. Full scans of Western blots shown in main figures. Cropped areas are marked by red color.

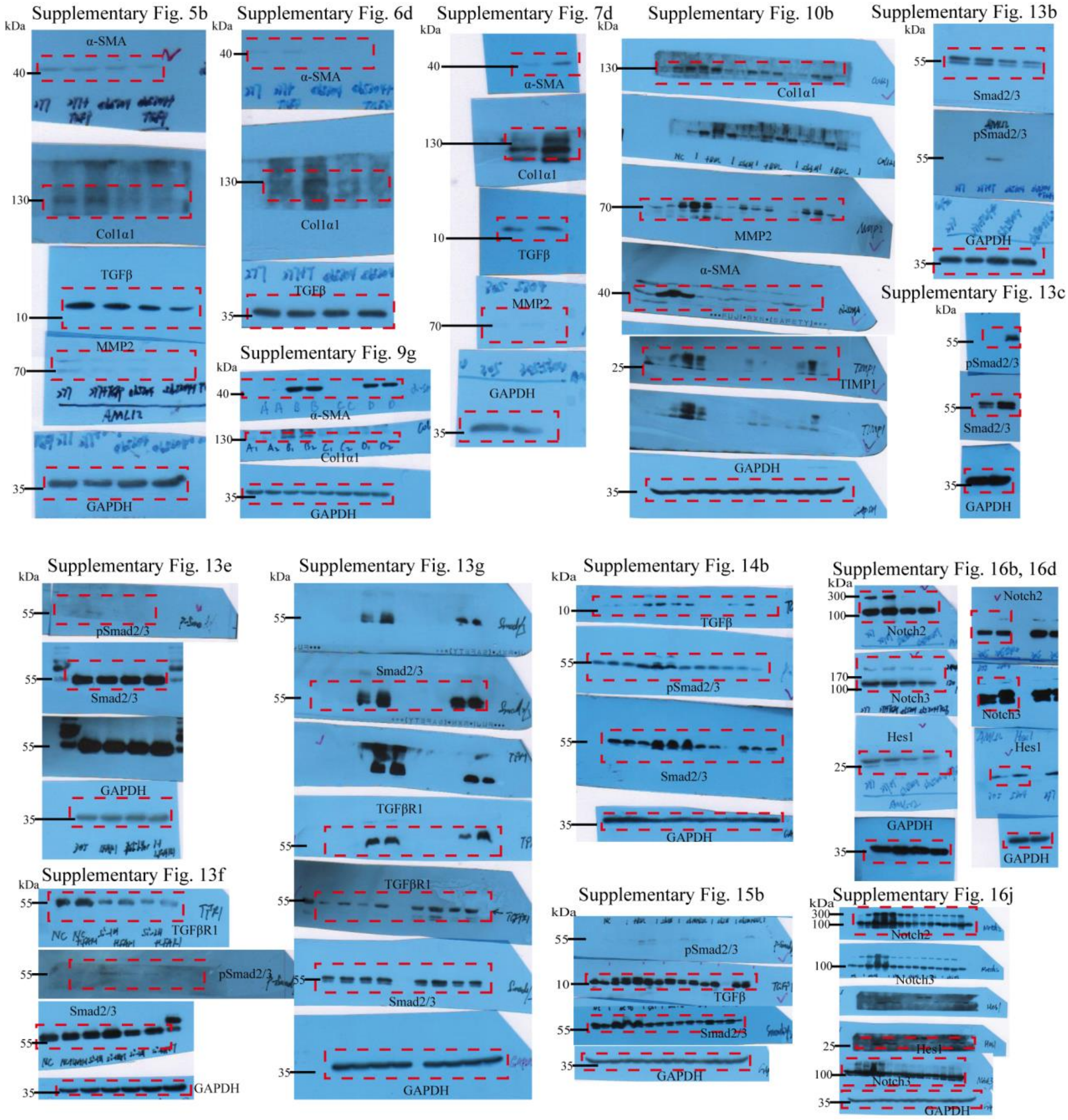

Supplementary Figure 19. Full scans of Western blots shown in Supplementary figures. Cropped areas are marked by red color.

Supplementary Table 1. Serum levels of ALT, AST in CCl_4-induced liver fibrosis model (mean ±SEM, n = 10)

Group	ALT (U/L)	AST (U/L)
NC group	38.3±6.4	47.4±12.5
NC+CCl_4 group	278.5±43.7*	323.6±61.2*
shLFAR1-3 group	35.3±7.2	38.8±8.3
shLFAR1-3+CCl_4 group	98.3±21.5#	151±43.8#

Data are presented as means ± s.e.m. *P* values were analysed by one-way ANOVA followed by *post hoc* comparison. **P*<0.05 compared with the NC group. #*P*<0.05 compared with NC+CCl_4 group.

Supplementary Table 2. Serum levels of ALT, AST in BDL-induced liver fibrosis model (mean ±SEM, n = 10)

Group	ALT (U/L)	AST (U/L)
NC group	40.3±8.6	62.4±11.7
NC+BDL group	236.3±65.3*	314.3±67.9*
shLFAR1-1 group	36.2±6.1	53.0±9.6
shLFAR1-1+BDL group	127.5±42.2#	137.2±37.5#
shLFAR1-3 group	38.7±7.3	58.6±15.5
shLFAR1-3+BDL group	116.6±34.3#	156.7±42.3#

Data are presented as means ± s.e.m. *P* values were analysed by one-way ANOVA followed by *post hoc* comparison. **P*<0.05 compared with the NC group. #*P*<0.05 compared with NC+BDL group.

Supplementary Table 3. Primers and Oligonucleotides, Related to Experimental Procedures

qRT-PCR primers for analysis of transcript levels

Gene symbol	Forward 5' - 3'	Reverse 5 '- 3'
NONMMUT013861	TGCTCTTACGGCTTCAATCA	GCTCACACCCATTCTCCCTA
NONMMUT069216	TGTGATGGTGTCTTGGTGGT	ATTGAAGGAGCCCAGTGTGA
ENSMUST00000147617	GTTGTGGTGATTGGAGCAG	CAGCGTGACCTATTCTGAGG
ENSMUST00000158992	CCAGGGAGGAATTGTGGTAA	TCTGTGTGTCATCTCTCAGTGG
NONMMUT040877	CAGGAGGAAGAAGCAGGTGT	GATTGGTTGGGTGGAGGTTT
NONMMUT042155	AGGCTTGGTGGCTCATACCT	GCTGGCTTGGAACACATTAGA
ENSMUST00000154817	GCTCTTTCATGGGAGCAACT	TCATTGCCTTTGGCTTTCTC
ENSMUST00000171651	GGTTCCTCGCTGATTCTTGA	TGGGATTGTGTCTCTGTCCA
NONMMUT043736	TGTGTGAGATGAGCGGTTTC	GGGTTGTGTAAGTGGGAGGA
MALAT1	AAATTGATGGCCTTTTCTGG	AGCTGGATCCTTGAGGTCAC
Actin	ATGCCACAGGATTCCATACCCAAGA	CTCTAGACTTCGAGCAGGAGATGG
GAPDH	GGCATGGACTGTGGTCATGAG	TGCACCACCAACTGCTTAGC
β-tubulin	CTGGGCTAAAGGCCAC	AGACACTTTGGGCGAG
lnc-LFAR1-1	GCCAGCACACTAAAGACGAG	GCAAAGGTGGAGGTCAGATT
lnc-LFAR1-2	GCTGGCCCCTTATTCCATGA	GCACGGTGTTAAACTGCTGG
lnc-LFAR1-3	CCAGCAGTTTAACACCGTGC	TCGGGAAAAGCGAACTCCTC
Col1α1	ATCGGTCATGCTCTCTCCAAACCA	ACTGCAACATGGAGACAGGTCAGA
Col1α2	CCTTTGTCAGAATACTGAGCAGC	GTAACTTCGTGCCTAGCAACA
Col3α1	TGCTCCAGTTAGCCCTGCAA	GGTCCTGCAGGCAACAGTGGTTC
Col4α5	CTCCCTTACCGCCCTTTTCTC	AGGCGAAATGGGTATGATGGG
CTGF	ATCCAGGCAAGTGCATTGGTA	GGGCCTCTTCTGCGATTTC
α-SMA	TCGGATACTTCAGCGTCAGGA	GTCCCAGACATCAGGGAGTAA
TIMP	TCCGTCCACAAACAGTGAGTGTCA	GGTGTGCACAGTGTTTCCCTGTTT
MMP2	GTGTTCTTCGCAGGGAATGAG	GATGCTTCCAAACTTCACGCT
TGF-β1	TGTGTTGGTTGTAGAGGGCAAGGA	TTTGGAGCCTGGACACACAGTACA
TβRI	GACAACATCAGGGTCTGGATCA	ACTTCTCCAAACCGACCTTTGC
Bcl2	GCTGGGATGCCTTTGTGGAACT	CAGAGACAGCCAGGAGAAATCAAAC
Bax	TTGCTGATGGCAACTTCAAC	GATCAGCTCGGGCACTTTAG
BAD	AGAGTATGTTCCAGATCCCAG	GTCCTCGAAAAGGGCTAAGC
TNF-α	CATCTTCTCAAAATTCGAGTGACAA	TGGGAGTAGACAAGGTACAACCC
MCP1	GTTAACGCCCCACTCACCTG	GGGCCGGGGTATGTAACTCA
Elastin1	CCACCTCTTTGTGTTTCGCTG	CCAAAGAGCACACCAACAATCAA
Fibrilin1	GCGCGGACGATACTTGAAGA	ACTGTCCGGCTGTCCTGAT
CyclinD1	TCAAGTGTGACCCGGACTG	ATGTCCACATCTCGCACGTC
c-Myc	GCGTTGGAAACCCCGCAG	AATAGGGCTGTACGGAGTCGT
Ankrd1	CACCCACCCACAGATTTGGT	TGCCTTCACCTTGGGACATC

AREG	TGGCATCGGCATCGTTATCA	TGTCATTTCCGGTGTGGCTT
Ptch1	ATGGCCTCGGCTGGTAACG	GCCAGTAGCCTTCCCCTTG
Gli1	AGATGATTCGGGTCTTTGGTCC	CCCTGGGACCCTGACATAAAG
Cerk	TTCGATACCACCCTCAACCT	CGGAAGAATGGATGTGGAAC
Papss1	CAGCAGCAGTGGAGTACAGG	GGTGACGTTGGTTGCTCTCT
Vimentin	TGCCAACCTTTTCTTCCCTG	TCTCTGGTCTCAACCGTCTT
Mapk1	CCTTCAGAGCACTCCAGAAAGT	ACAACACCAAAAAGGCATCC
KLF6	CGCACTCACACAGGAGAAAA	GTATGCTTTCGGAAGTGTCT
E-cadherin	GACAGAAACGAGACTGGGTCA	CCGGTGATGCTGTAGAAAACC
N-cadherin	CAAAGGCAGAAGAGAGACTGG	ATGAAGATGCCCGTTGGAGGC
ZO-1	GCCGCTAAGAGCACAGCAA	TCCCCACTCTGAAAATGAGGA
Smad2	CTTGGCTGTCCTCATACACGAA	CCGAGTCTCCTGTTCCCGTA
Smad3	GTTGGACGAGCTGGAGAAG	GTAGTAGGAGATGGAGCAC
Notch2	TGACTGTTCCCTCACTATGG	CACGTCTTGCTATTCCTCTG
Notch3	TTGTCTGGATGGAAGCCCATGT	ACTGAACTCTGGCAAACGCCT
hey2	GTGCGCCTTGTCTCTCATCT	ATAGGCGACATGGGGTTGAC
Hes1	CTCCCGGCATTCCAAGCTAG	AGCGGGTCACCTCGTTCATG
Jag1	GGGAGAGTGATACTTGATGGG	CTCATTGTGGCTTTTGTGGAG
Jag2	AACCTGATTGGCGGCTATTAC	CGTACTCTAGTTCGCAATGGC

RACE primers for lnc-LFAR1

gene specific primer	Sequence 5' - 3'
3' OUTER PRIMER	GATTACGCCAAGCTTCTGACCTCCACCTTTGCTGGCCCCT
5' OUTER PRIMER	GATTACGCCAAGCTTCCATCCAACAGCAGTGGGCTCGGGA
5' INNER PRIMER	GATTACGCCAAGCTTAAGCGAACTCCTCCTGCAGCCGTGT

Cloning primers for lnc-LFAR1

Name	Sequence 5' - 3'
lnc-LFAR1 5' BamHI F	CGCGGATCCACATGGGAGACAGGGTTTCT
lnc-LFAR1 5' BamHI F	CGCGGATCCAGACAGGGTTTCTCTGTATAGC
lnc-LFAR1 5' BamHI R	CGCGGATCCGAAATAAGCGGTTTAATAGGATCACC

siRNAsequences

Name	Forward 5' - 3'	Reverse 5' - 3'
negative control	GUUCUCCGAACGUGUCACGTT	CGUGACACGUUCGGAGAACTT
lnc-LFAR1-1	GGUCACGAUUCAUCUGAAATT	UUUCAGAUGAAUCGUGACCTT
lnc-LFAR1-2	GGACCUCAUCUGUAAUGAATT	UUCAUUACAGAUGAGGUCCTT
lnc-LFAR1-3	GAUCCUAUUAAACCGCUUATT	UAAGCGGUUUAAUAGGAUCTT
TβRI-1	GAUGGUCUUUGCUUUGUCUTT	AGACAAAGCAAAGACCAUCTT
TβRI-2	CCAGGACCAUUGUGUUACATT	UGUAACACAAUGGUCCUGGTT
TβRI-3	GUUGGUGUCAGAUUAUCAUTT	AUGAUAAUCUGACACCAACTT

shRNAsequences

Name	Sequence 5' - 3'
sh-LFAR1-1 Forward	GATCCCCGGTCACGATTCATCTGAAATTCAAGAGATTTCAGATGAATCGTGACCTTTTTA
sh-LFAR1-1 Reverse	AGCTTAAAAAGGTCACGATTCATCTGAAATCTCTTGAATTTCAGATGAATCGTGACCGGG
sh-LFAR1-2 Forward	GATCCCCGGACCTCATCTGTAATGAATTCAAGAGATTCATTACAGATGAGGTCCTTTTTA
sh-LFAR1-2 Reverse	AGCTTAAAAAGGACCTCATCTGTAATGAATCTCTTGAATTCATTACAGATGAGGTCCGGG
sh-LFAR1-3 Forward	GATCCCCGATCCTATTAAACCGCTTATTCAAGAGATAAGCGGTTTAATAGGATCTTTTTA
sh-LFAR1-3 Reverse	AGCTTAAAAAGATCCTATTAAACCGCTTATCTCTTGAATAAGCGGTTTAATAGGATCGGG
shSmad2-1 Forward	GATCCCCCCCATCAAAGACTCGCTGTTTCAAGAGAACAGCGAGTCTTTGATGGGTTTTTA
shSmad2-1 Reverse	AGCTTAAAAACCCATCAAAGACTCGCTGTTCTCTTGAAACAGCGAGTCTTTGATGGGGGG
shSmad2-2 Forward	GATCCCCCCACTGTAGAAATGACAAGTTCAAGAGACTTGTCATTTCTACAGTGGTTTTTA
shSmad2-2 Reverse	AGCTTAAAAACCACTGTAGAAATGACAAGTCTCTTGAACTTGTCATTTCTACAGTGGGGG
shSmad3-1 Forward	GATCCCCGGCCATCACCACGCAGAACTTCAAGAGAGTTCTGCGTGGTGATGGCCTTTTTA
shSmad3-1 Reverse	AGCTTAAAAAGGCCATCACCACGCAGAACTCTCTTGAAGTTCTGCGTGGTGATGGCCGGG
shSmad3-2 Forward	GATCCCCAGACAGACAGTGACCAGCATTCAAGAGATGCTGGTCACTGTCTGTCTTTTTTA
shSmad3-2 Reverse	AGCTTAAAAAAGACAGACAGTGACCAGCATCTCTTGAATGCTGGTCACTGTCTGTCTGGG
Negativecontrol Forward	GATCCCCGTTCTCCGAACGTGTCACGTTCAAGAGACGTGACACGTTCGGAGAACTTTTTA
negative control Reverse	AGCTTAAAAAGTTCTCCGAACGTGTCACGTCTCTTGAACGTGACACGTTCGGAGAACGGG

Primers for ChIP qRT-PCR

Locus	Forward 5' - 3'	Reverse 5 '- 3'
lnc-FLAR1(-355--232)	GCTGCTAAGACAACCCAGAA	TTTAGCGGCTGTTACCTTCCC
lnc-FLAR1(-959--834)	CCTTTTGCTGCCAGGGGTAA	ACCATGGTTCCTTCACGGTT
lnc-FLAR1(-1279--1152)	GTAGCCACGTGAACGGTGAG	CGGACCAATCCCACAACTCC
lnc-FLAR1(-1762--1599)	TCATCTCTCAGCCTCCGTCA	CTAGATGTTGCAGGGAGGGG
Col1α1(-424--253)	CCAGGAGGACCTTTTCCCAA	GTGCTGTCACTGGAGTGTGG
Col1α1(-730--640)	GGATGTCAAAGGTCTCCCCA	GGGTGCCTATCTGTTCTGCC
Col1α1(-1455--1380)	GACTCCCTGCTTCCACGTTT	TTGCAGGGCCCATAGACATC
Col1α1(-1969--1869)	GCTTCGTGGCATTCTACCCT	TTCCAAAGGATGCCCCACTC
Col1α2(-340--230)	AGCCCACGTAGGTGTCCTAA	GCTTTCGAGGGGGAACTCTG
Col1α2(-1343--1243)	TTCCTCACCGGGAAGTCGAA	TCACAGCAGACACAGCATCTT
α-SMA(-549--314)	AGGAGAGTGAGCAGGCTTCATT	AGTGAGGATTAACCAGCCTGT
α-SMA(-1147--1242)	AACTATGCATGCGCTCAGGT	TAGGGAAACCCCAGGGTGAA
α-SMA(-1929--1998)	GAGGAATGTGCAAACCGTGC	CAACTGCTCAAATGCCCAGAC
CTGF(-39-+81)	GAATGTGAGGAATGTCCCTGTT	CTTGGAGAGAAGAGCTGTGTGA
CTGF(-1090--1059)	CAACACACGAGCAGGGGATA	AATAGCTTGCAGGCTCGTGG
TGFβ1(-231--93)	TCACCGGCTTTAGTAGTGCTC	GGGGGCACTGTCTTCATCT
TGFβ1(-1263--1089)	TGGACTTTGTTCTGTGGCCC	GAAACCACTGGAGACCTCGC
TGFβ1(-1984--1915)	AGAGTCTCAGAACATAGTCCAGC	GAAGGGTGACATTTTGGCACA
MMP2(-440--305)	GTTTGGAGAAGGAAGGCTGGT	AGAAACAAGAGGGTCCCAACC
MMP2(-843-729)	CCAACTCTGTTCAGGCAGGT	CAGGGGCCAGCAAGGATAAT
MMP2(-1517--1389)	CCCAGCTCAGGTCCTTGTTT	AGGGATTCACGGTTGTCACC
TIMP1(-630--440)	TAGGACTCCAGGGTCAGGAAG	AGCCTAGGTACCCCAAACCT
TIMP1(-291--132)	AGTTTGTCACCCTCTGACACC	AAGCTTTGTGCCTCTCAGGTT
TIMP1(-1909--1837)	GGCTCATAGAAGAGGCGAGAC	GCTGAGTAACTAGGCGGCAG
Notch2(-371--300)	TTTGATGTTGGGCGCTTCAG	GGTTTCCCGCAGAAAGAAGC
Notch2(-1123--1014)	CACCCATTTGCACTTGCTGAA	ACACGGGGAAGTCTTTATGGC
Notch2(-1965-1890)	GGTAACACCATGGGTGAACAAA	GGCAATTTCTGCTTGTGCCAT
Notch3(-234--165)	TTGCAGACCTCGGTACACTC	GATACCTGTCACGTCACGCA
Notch3(-1083--1014)	CTCCATCACTAGGAGACCAAAGG	GTGTCTGTGTATGCCCTTCCA
Notch3(-1908--1765)	AGAACCTGGGGTTTCCAGTG	GGGATCCAGTCTTCGGTCCA
Hes1(-223--60)	TTGACGTTGTAGCCTCCGGT	AACGGCTCGTGTGAAACTTCC
Hes1(-1199-1007)	CAGCTGCTATTTACCTTCTTGGC	AGCACGTGCCAGGATGTTTT
Hes1(-1609--1522)	AAGTGCGGTCAGGCATCTC	ATCTGAGCGTGGCCGAAAC
Smad2(-222--80)	TCAAGGAGCACACGCATAGG	TCCGTGCGGTTGGTATTAGG

Smad2(-1076-1002)	TGGTGGTGCTGGGGTTAAAA	GCAGAGGATAGAGCTTCCCG
Smad2(-1853--1733)	TTCAGCTCGTCTTGACCCAC	AAAGGGAATAGGGGGCAACC
Smad3(-198--7)	GAAGGAAAGTCCAACCCCCA	GCTGCGTGAAACGTAGACTTG
Smad3(-1271--1038)	ACGGATTTGGGGCGTTACAT	ATAGGGCTTCGTAAAGCGCA
Smad3(-1952-1778)	TGCTACTGGCCCTAGAACTGT	GAATCAACACTGGCCTCCACT
Gapdh intron	ATCCTGTAGGCCAGGTGATG	AGGCTCAAGGGCTTTTAAGG
PAI1	TCCAATCCAGCCATCAGCAC	CAGAGGGCATGAAATGTGCC

Primers for constructing the EGFP tagged expression vectors

Primer name	Forward 5'-3'	Reverse 5'-3'
lnc-LFAR1 ORF	CCCAAGCTTTGCAGGAGGAGTTCGCTTTT	CGCGGATCCGAAATAAGCGGTTTAATAGGATCACC
lnc-MALAT1 ORF	CCCAAGCTTGCGCATGTACGTTTGAAGGC	CGCGGATCCTGCTGGCATCCAAAGTTGTC
GAPDH (1st exon)	CCCAAGCTTCCCTTAAGAGGGATGCTGCC	CGCGGATCCACTGTGCCGTTGAATTTGCC

Primers for constructing the luciferase reporter plasmids

Name	Forward 5' - 3'	Reverse 5 '- 3'
lnc-LFAR1 WT	CGGGGTACCCTCCATGTACTGGTCGGTCC	CCGCTCGAGCTCAACACCTGGTTATATCCCAC
lnc-LFAR1 Mut1	CCCGAGGGAGGGGCCCACCACAAGCACACTGAG	CTCAGTGTGCTTGTGGTGGGCCCCTCCCTCGGG
lnc-LFAR1 Mut2	CCGTTCACGTGGCTACAGCAAGAGGACACACGG GTGGACAAC	GTTGTCCACCCGTGTGTCCTCTTGCTGTAGCCA CGTGAACGG
